# Effects on Sleep Quality of Physical Exercise Programs in Older Adults: A Systematic Review and Meta-Analysis

**DOI:** 10.3390/clockssleep5020014

**Published:** 2023-03-23

**Authors:** Lilian Solis-Navarro, Olga Masot, Rodrigo Torres-Castro, Matías Otto-Yáñez, Carles Fernández-Jané, Mireia Solà-Madurell, Andrea Coda, Erika Cyrus-Barker, Mercè Sitjà-Rabert, Laura Mónica Pérez

**Affiliations:** 1 Programa de Doctorat, Facultat Ciències de la Salut Blanquerna, Universitat Ramon Llull, 08025 Barcelona, Spain; 2 Blanquerna School of Health Sciences, Global Research on Wellbeing (GRoW), Universitat Ramon Llull, 08025 Barcelona, Spain; 3 Escuela de Kinesiología, Facultad de Salud y Odontología, Universidad Diego Portales, Santiago 8370109, Chile; 4 Department of Nursing and Physiotherapy, University of Lleida, 25198 Lleida, Spain; 5 Health Care Research Group (GRECS), Biomedical Research Institute of Lleida, 25198 Lleida, Spain; 6 Department of Physical Therapy, Faculty of Medicine, University of Chile, Santiago 8380453, Chile; 7 Institut d’Investigacions Biomèdiques August Pi i Sunyer (IDIBAPS), 08036 Barcelona, Spain; 8 Kinesiology School, Universidad Autónoma de Chile, Santiago 7500912, Chile; 9 Tecnocampus, Universitat Pompeu Fabra, Mataró-Maresme, 08302 Barcelona, Spain; 10 School of Health Sciences, College of Health, Medicine and Wellbeing, University of Newcastle, Ourimbah, NSW 2258, Australia; 11 Equity in Health and Wellbeing Research Program, Hunter Medical Research Institute, New Lambton Heights, NSW 2305, Australia; 12 Physical Therapy Department, Santa Paula University, San José 11803, Costa Rica; 13 Research on Aging, Frailty and Care Transitions in Barcelona (RE-FiT), Parc Sanitari Pere Virgili and Vall d’Hebron Institute (VHIR), 08023 Barcelona, Spain

**Keywords:** older adults, obstructive sleep apnoea, exercise, sleepiness, sleep quality

## Abstract

Background: Given the beneficial effects of exercise in different populations and the close relationship between healthy ageing and sleep quality, our objective was to determine if physical exercise delivered through a structured program improves sleep quality in older adults. Methods: Embase, PubMed/MEDLINE, Web of Science, and Cochrane Register of Clinical Trials (CENTRAL) were searched to 15 January 2023. Studies that applied physical exercise programs in older adults were reviewed. Two independent reviewers analysed the studies, extracted the data, and assessed the quality of evidence. Results: Of the 2599 reports returned by the initial search, 13 articles reporting on 2612 patients were included in the data synthesis. The articles used interventions based on yoga (*n* = 5), multicomponent exercise (*n* = 3), walking (*n* = 2), cycling (*n* = 1), pilates (*n* = 1), elastic bands (*n* = 1), and healthy beat acupunch (*n* = 1). In the intervention group, we found significant improvement in Pittsburgh sleep quality index of −2.49 points (95% CI −3.84 to −1.14) in comparison to the control group (*p* = 0.0003) and sleep efficiency measured with objective instruments (MD 1.18%, 95% CI 0.86 to 1.50%, *p* < 0.0001). Conclusion: Our results found that physical exercise programs in older adults improve sleep quality and efficiency measured with objective instruments.

## 1. Introduction

The number of older people is growing worldwide. Currently, about 10% of the global population is over 65 years old [1], and it is projected that between 2015 and 2050, the proportion of the world’s population over 60 years old will nearly double from 12 to 22% [2]. The challenge for this population is to achieve healthy ageing [2]. In addition, the clinical guidelines widely recommend physical activity as a central component of healthy ageing [3,4]. Regular exercise leads to ageing actively and satisfactorily because it is associated with physical, functional, psychological, and cognitive improvement [5].

Sufficient lifetime physical activity, healthy food habits, and significant sleep quality are major contributors to healthy ageing [6,7]. On the other hand, poor sleep quality and insufficient habitual physical activity behaviour are reported to deteriorate health along with the ageing process [8]. Furthermore, poor sleep quality has been related to negative health outcomes, and an increase in the speed of multimorbidity development [9] and these deteriorations have been associated with losses in cognitive and functional capacity [10,11].

Sleep disorders, such as altered sleep duration and increased sleep fragmentation, are common among older adults [12]. The most frequent sleep disorders that have been described in older adults are difficulty initiating and maintaining sleep and waking up early in the morning [13]. These sleep disorders in this population may be attributed to a lack of physical exercise, poor sleep habits, and inactive lifestyles with repetitive daily routines [14].

Poor sleep quality in older adults is expressed, among other ways, as insomnia, a sleep–wake disorder characterised by difficulty initiating or maintaining sleep or both, and older adults are more likely to be prescribed medication for insomnia treatment [15]. Medication classes commonly used to treat insomnia—including benzodiazepines and z-drugs (such as zolpidem)—can have adverse effects, including cognitive and memory impairment, rebound insomnia upon cessation, and increased risk of motor vehicle accidents, falls, dependency, and addiction [16,17]. Thus, these medications may not be appropriate for use in older adults due to unfavourable risk–benefit ratios [18], particularly for those with a history of falls or fractures [19]. Furthermore, individuals receiving insomnia treatment had an increased risk of falls and mortality and higher healthcare resource utilisation and costs than matched beneficiaries without sleep disorders [18].

Exercise is a widely accepted approach adopted to improve physical function, cardiovascular health, and mental health, and it may also be beneficial for sleep [20,21,22,23,24]. The exercise can be performed in different environments, such as rehabilitation centres, gyms, public parks, or at home [20]. The exercise program is considered home-based if the physical exercise is performed in an informal and flexible place such as the individual’s house. Each program should have clear goals, including monitoring, follow-up visits, calls from health professionals, or self-monitoring diaries [25].

Given the beneficial effects of exercise in different populations and the close relationship between healthy ageing and sleep quality, our objective was to determine if physical exercise delivered through a structured program improves sleep quality in older adults.

## 2. Methods

### 2.1. Protocol and Registration

A systematic review was reported according to the Preferred Reporting Items for Systematic Reviews and Meta-Analyses (PRISMA) guidelines [26]. The review was registered in the International Prospective Register of Systematic Reviews (PROSPERO) CRD42022369802.

### 2.2. Criteria for Considering Studies in This Review

Randomised controlled trials (RCTs) that included older adults (>65 yr) [27] performing a structured exercise program were searched. The included studies aimed to determine the effects of exercise on sleep quality. The search strategy was based on the PICO model (population: older adults; intervention: exercise program; control: no intervention or placebo; and outcome: sleep quality, sleepiness, apnoea–hypopnoea index) [28].

### 2.3. Search Strategies and Data Resources

The Embase, PubMed/MEDLINE, Web of Science, and Cochrane Register of Clinical Trials (CENTRAL) were reviewed on 23 October 2022 and updated on 15 January 2023. Manual searches with the following terms were conducted: ((elderly) OR (older people) OR (older adults) OR (ageing)) AND ((Exercise) OR (physical training) OR (physical activity) OR (resistance training) OR (aerobic) OR (gym) OR (gymnastic) OR (strength) OR (balance)) AND ((sleep quality) OR (sleepiness) OR (apnea-hypopnea index)) (supplementary material 1). No language or publication restrictions were imposed.

The terms selected were combined using Boolean logical operators (OR, AND, NOT). Additionally, a manual search of the references included in the selected articles was conducted. All references were analysed using Rayyan web software [29].

### 2.4. Reviewing Procedure and Data Extraction

The selected articles were reviewed independently by investigators with experience in meta-analysis and training in the literature review. First, the titles and abstracts of all identified studies were reviewed in pairs by four investigators (LSN-MOY-OM-RTC). Studies deemed irrelevant based on the title and abstract were excluded. Any possible disagreements were resolved by a third reviewer (MSR). Second, the first step’s full-text versions of the articles selected were reviewed and rechecked against the eligibility criteria (LSN-RTC). Again, any potential disagreements were resolved by a third reviewer (MSR). There were no disagreements between the investigators. Finally, study authors were contacted as required to determine study eligibility.

Two authors (OM-RTC) extracted the data using a standardised protocol and reporting forms. The following information was extracted from each included study: design, population characteristics, and exercise program characteristics (type of exercise, setting, duration, intensity, frequency). If some relevant data were not in the article, the author was contacted to request the information.

### 2.5. Methodological Quality Assessment

The primary articles’ methodological quality was assessed using the Cochrane Collaboration tool to assess the risk of bias (the Cochrane Handbook for Systematic Reviews of Interventions) [28]. The tool included seven items: generation of a random sequence, allocation concealment, blinding of participants and personnel, blinding of outcome assessment, completeness of outcome data, selection of reports, and other biases. For each item, the risk of bias for the study was rated according to three categories: low, high, or unclear risk of bias. Two reviewers (CFJ-MSM) independently assessed the risk of bias in the studies. A third author (RTC) was consulted for discrepancies that could not be resolved.

### 2.6. Data Synthesis and Analysis

A narrative synthesis was used to summarise the characteristics of studies, including population and intervention characteristics. Summaries of the association between the outcomes for each study in terms of mean differences (MDs) or standard mean differences (SMDs) using Review Manager 5 (RevMan, Copenhagen, Denmark: The Nordic Cochrane Centre, The Cochrane Collaboration, 2014) were reported. Absolute values and obtained combined measures of the effect of each primary outcome through meta-analysis with a random-effect model due to the expected heterogeneity between studies were compared [28]. Statistical heterogeneity was measured with the I^2^ statistic and classified as low (I^2^ < 25%), moderate (I^2^ = 25–50%), or high (I^2^ > 50%) [28]. A sub-analysis was carried out by type and duration of training and setting for the main outcome. Additionally, the overall certainty of evidence was assessed independently by two reviewers (RTC, LSN) using the GRADE approach [30]. Disagreements were solved by consensus. Publication bias was assessed by visualising a funnel plot and Begg’s and Egger’s tests for the possible existence of study bias using the Jamovi software (version 2.3) [31].

## 3. Results

### 3.1. Study Selection

**Figure 1:**
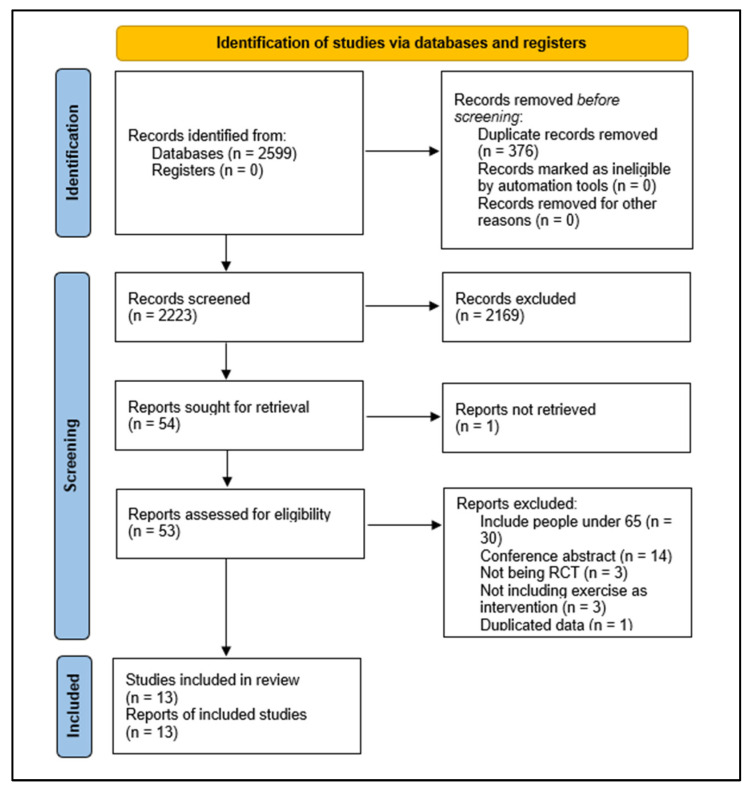
Study selection process.

The initial search yielded 2599 potential studies. In total, 376 duplicate records were deleted. We screened 2223 titles and abstracts and excluded 2169 records that did not meet the inclusion criteria. Fifty-four of these were assessed as full text. Of these, 30 were excluded for including people under 65 years old, 14 for being conference abstracts, 3 for not being RCTs, 3 for not including exercise as intervention, and 1 for duplicated data. Ultimately, 13 studies met the criteria for eligibility and were included in the review [32,33,34,35,36,37,38,39,40,41,42,43,44]. The flow chart of the study selection process is shown in Figure 1.

### 3.2. Characteristics of the Included Studies

Three studies were conducted in Taiwan [32,41,42], two in the USA [38,40] and Turkey [34,39], and one in Korea [43], Spain [44], the UK [37], Tunisia [36], Canada [35], and Japan [33]. All studies were published after 2010. The characteristics of included studies are in Table 1.

### 3.3. Participants

In total, 2612 older adults were analysed (1304 in the intervention group and 1308 in the control group). Sample sizes varied between 21 [37] and 1635 [40] participants. The studies included 864 (33.8%) males and 1693 (66.2%) females, with mean age varying between 68 ± 2 [37] and 80.1 ± 6.4 [41] years. One study did not report sex distribution [32]. The body mass index (BMI) varied between 23.4 ± 17.7 [44] and 31.5 ± 3.6 [39] kg/m^2^ (Table 1). Most of the studies were conducted in older adults living in the community [35,36,37,38,40,42,43,44], and a third of the studies were conducted in older adults living in nursing homes or assisted living facilities [12,32,34,39]. Furthermore, a study was carried out on carers of people with dementia [33].

### 3.4. Characteristics of Training

Five articles used interventions based on yoga [32,36,38,39,43], three in multicomponent exercise [34,40,44], two in walking [33,35], one in cycling [37], one in pilates [39], one in elastic bands [41], and one in healthy beat acupunch [42]. The duration of the programs varied between 8 and 12 weeks [33,36,38,39,43,44], between 12 weeks and 12 months [32,34,37,41,42], and more than 12 months [35,40]. The details of the training programs are shown in Table 2.

### 3.5. Methodological Quality Assessment

**Figure 2:**
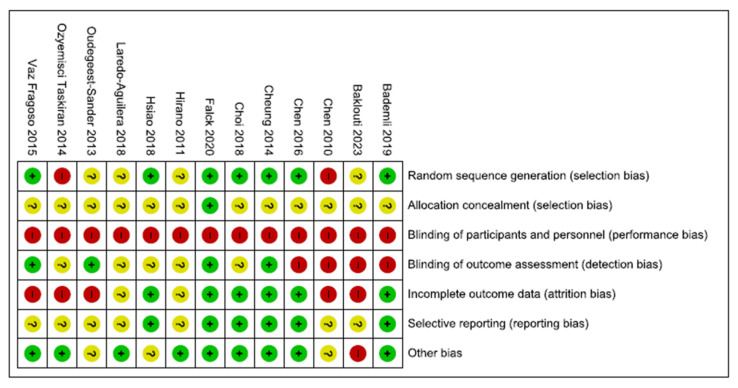
Risk of bias summary. Legend: Red (−) = high risk of bias; Yellow (?) = unknown risk of bias; Green (+) = low risk of bias.

**Figure 3:**
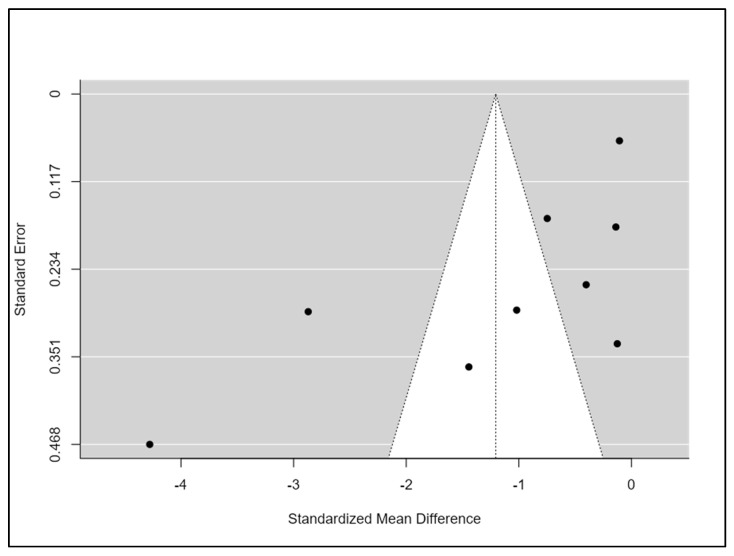
Risk of bias summary.

All studies had a high or unclear risk of bias in at least one domain. The majority of studies claimed to be randomised. However, only half of them explain how the randomisation was conducted [34,35,38,40,41,43]. No study reported that participants and personnel were blinded. However, this is a common feature in non-pharmacological studies, particularly in rehabilitation studies, in which it is complex to blind professionals or patients due to the nature of the intervention. One-third of studies reported that researchers and outcome assessments were blinded [35,37,38,40]. Five studies had a low risk of bias on attrition rates and selection reporting [34,35,38,41,43]. Finally, the majority of the studies had a low risk of other potential sources of bias (Figure 2). Funnel plots and Begg’s and Egger’s tests indicated a publication bias for the estimation of the effect of exercise on sleep quality (Begg = 0.08; Egger = 0.009) (Figure 3).

### 3.6. Main Findings

**Figure 4:**
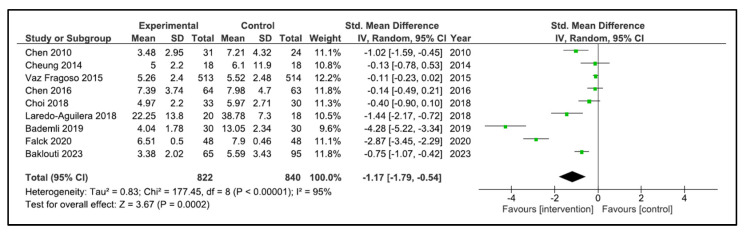
Forest plot for sleep quality.

**Figure 5:**
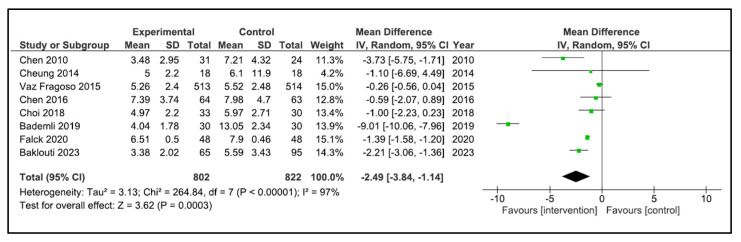
Forest plot for PSQI.

Sleep Quality: A total of 13 studies reported sleep quality [32,33,34,35,36,37,38,39,40,41,42,43,44]. The instruments used were the PSQI [32,34,35,36,38,40,41,42,43], the Oviedo sleep questionnaire [44], the quality sleep score [33], the sleep dimension of Nottingham health profile (*n* = 1) [39], and an accelerometer/actigraphy [35,37]. A meta-analysis was pooled with nine studies [32,34,35,36,38,40,41,43,44]. These studies compared 822 patients in the intervention group (IG) versus 840 in the control group (CG). Older people in the IG had an SMD of −1.17 points (95% CI −1.79 to −0.54) in comparison to CG (*p* = 0.0002) (Figure 4). The heterogeneity of the comparison was high (I^2^ = 95%). If we analysed only the studies with PSQI [32,34,35,36,38,40,41,43], there were 802 patients compared in the IG versus 822 in the CG. Older people in the IG had an MD of −2.49 points (95% CI −3.84 to −1.14) in comparison to CG (*p* < 0.0001) (Figure 5). The heterogeneity of the comparison was high (I^2^ = 98%). The certainty of evidence, according to the GRADE methodology, was moderate. Although four studies [33,37,39,42] could not be pooled due to the way they reported the results, it is important to note that three of the four reported an improvement in sleep quality after the intervention [33,39,42].

**Figure 6:**
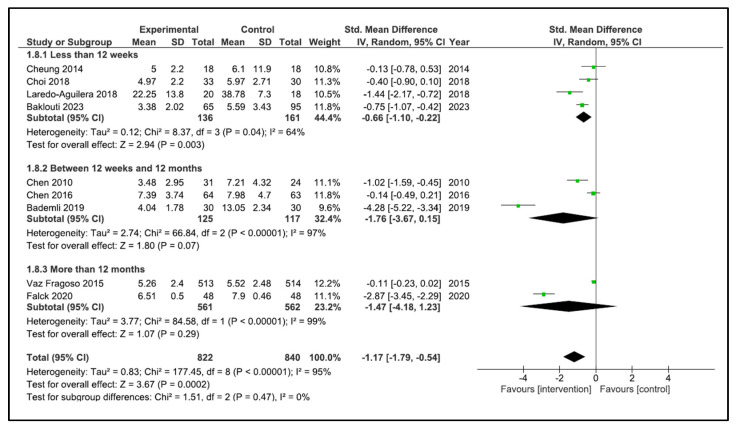
Forest plot for sleep quality by duration time.

**Figure 7:**
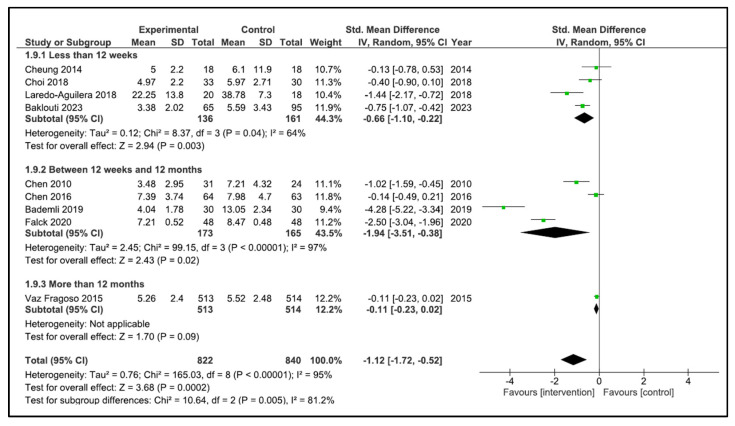
Forest plot for sleep quality by duration time including intermediate assessment points.

When performing the analysis by time duration, the programs with duration less than 12 weeks improve the sleep quality (SMD −0.66, 95% CI −1.10 to −0.22, *p* = 0.003) with a high heterogeneity (I^2^ = 64%). However, the programs carried out between 12 weeks and 12 months (SMD −1.76, 95% CI −3.67 to 0.15, *p* = 0.07) and more than 12 months (SMD −1.47, 95% CI −4.18 to 1.23, *p* = 0.29) did not improve sleep quality. The heterogeneity was high in both cases (I^2^ = 97, and I^2^ = 95%, respectively) (Figure 6). If we analysed by time duration, but including intermediate assessment points of high-duration programs, the programs between 12 weeks and 12 months improve the sleep quality (SMD −1.94, 95% CI −3.51 to −0.38, *p* = 0.02) with a high heterogeneity (I^2^ = 97%), and the programs of more than 12 months (SMD −0.11, 95% CI −0.23 to 0.02, *p* = 0.09) did not improve sleep quality (Figure 7).

**Figure 8:**
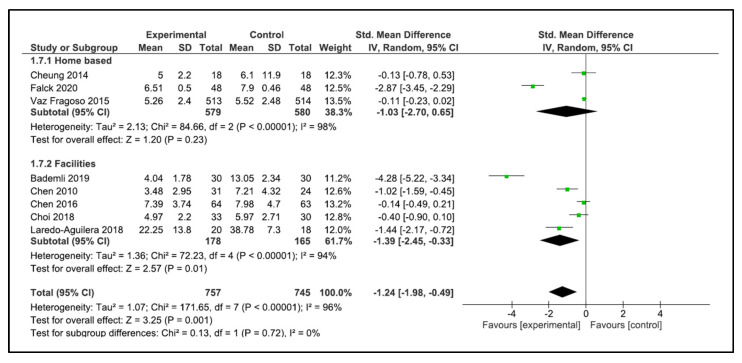
Forest plot for sleep quality by setting.

When performing the analysis by setting, the programs carried out at home did not improve sleep quality (SMD −0.95, 95% CI −1.91 to 0.02, *p* = 0.06). However, the programs carried out in the facilities showed a significant improvement in sleep quality (SMD −1.39, 95% CI −2.45 to −0.33, *p* = 0.01) (Figure 8). No significant differences were found when performing the analysis by type of training.

**Figure 9:**
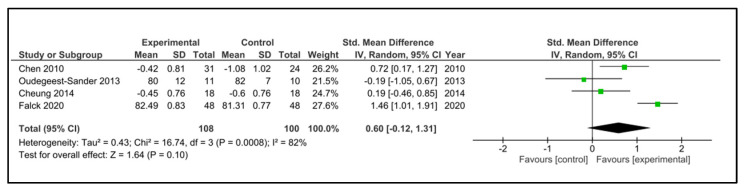
Forest plot for sleep efficiency. Subjective and objective measures.

**Figure 10:**
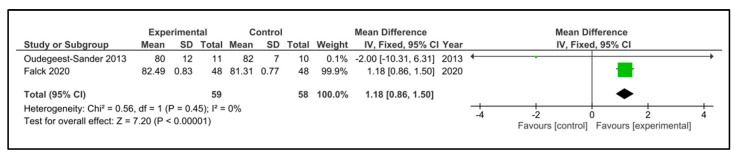
Forest plot for sleep efficiency. Objective measures.

Sleep efficiency: Five studies examined sleep efficiency [32,35,37,38,42]. The instruments used were accelerometry/actigraphy [35,37] and a specific domain of PSQI [32,38,42]. These studies compared 108 participants in the IG versus 100 participants in the CG. The heterogeneity was moderate (I^2^ = 38%). Older people in the IG had no change after intervention (SMD 0.60, 95% CI −0.12 to 1.31) in comparison to CG (*p* = 0.10). The heterogeneity of the comparison was high (I^2^ = 82%) (Figure 9). If we analysed only the studies with an objective measure of sleep efficiency [35,37], there were 59 older people compared in the IG versus 58 in the CG. Older people in the IG had a higher sleep efficiency post-intervention (MD 1.18%, 95% CI 0.86 to 1.50%) in comparison to CG (*p* < 0.0001) (Figure 10). The heterogeneity of the comparison was low (I^2^ = 0%). The certainty of evidence, according to the GRADE methodology, was low.

Sleepiness: Only one study examined the Epworth sleepiness scale (ESS) post-intervention [40]. No significant benefits of exercise programs were shown for prevalent cases of poor sleep quality or sleep–wake behaviours that were evaluated using the ESS.

## 4. Discussion

This systematic review provides new information about the effects of exercise on sleep quality measured exclusively in the older adult population, showing that exercise improves sleep quality, particularly in facilities programs, and improves sleep efficiency measured with objective instruments. However, these results must be analysed with caution because there is a high heterogeneity in interventions and baseline characteristics of the population.

### 4.1. Effects on Sleep Quality

Our meta-analysis reports that exercise improves sleep quality. One possible explanation could be that physical activity regulates sleep patterns [34]. With increasing age, there are substantial changes in sleep quality and quantity, including slow-wave sleep, spindle density, and sleep continuity [45]. Furthermore, it seems that for older adults with sufficient mobility and no pre-existing disease, a structured physical activity program may help reduce insomnia [46].

Poor sleep quality is closely related to mental health problems in older adults. Although this research did not explore anxiety or depression as an outcome, exercise in older adults has already been reported to have a positive effect in reducing depression and anxiety [32]. Another aspect to consider is whether the improvement in physical capacity can reduce the use of medications. Although some reports show no statistically significant association between the change in physical capacity and the use of benzodiazepines and z-hypnotics, a decrease in psychotropic drugs and antidepressants has been observed when physical capacity improves in community-dwelling older adults [47]. The high use of psychotropics, like benzodiazepines, may have detrimental effects on older adults (i.e., risk for fractures, falls, and premature mortality, among others) [48,49,50], and it is reported that only about half of the older population who take psychotropics respond to these medications [51].

The mechanisms that explain the improvement in sleep quality due to exercise in older adults are still not completely clear. One possible explanation is that exercise training provides a benefit on sleep quality by decreasing the sleep latency and the use of sleep medication in older adults [52]. Another possible mechanism is that during sleep there is a fluid accumulation in the neck, which causes an increase in the pressure on the upper airway, which can cause the onset of obstructive sleep apnoea. After exercise, there is a significant reduction in the amount of fluid in the neck [53,54]. Other proposed mechanisms include body temperature changes by effects on adenosine levels, cytokine concentration changes, increased energy consumption/metabolic rate, changes in mood/anxiety symptoms, growth hormone secretion, improved fitness level, and body composition change, among others [54,55,56,57,58].

### 4.2. Sleep Quality Assessment

Sleep quality is a concept that includes quantitative aspects of sleep and more subjective aspects, such as “depth” or “restfulness” of sleep [59]. The most used instrument was PSQI, an index created in the psychiatric field [59]. In our meta-analysis, we observed a change of −2.49 units in the PSQI. Limited studies evaluate the minimal clinically important difference (MCID) for this instrument, suggesting a change between 1.54 and −3 [60,61]; however, these data have not been determined for older adults. Due to the lack of universal acceptance of an MCID for PSQI, any statistically significant effect between groups at the end of treatment was considered important in this study.

Sleep is a complex phenomenon, and the literature suggests that objective and subjective indices measure different components of sleep in older adults [62]. Specifically, subjective sleep quality, according to the PSQI, appears to be poorly correlated with sleep measured by actigraphy or polysomnography, and the lack of relationship between objective and subjective sleep measures appears to be unrelated to age, gender, education, or cognitive status [35]. Therefore, both types of evaluation should complement each other. Based on the evidence, an objective and subjective assessment of sleep quality is suggested, particularly because among the selected studies, only one [35] concluded that a multimodal lifestyle could improve subjective, but not objective, sleep in older adults with mild cognitive impairment and poor sleep.

One of the outcomes analysed was sleep efficiency, which was objectively reported through accelerometry and actigraphy [35,37] and subjectively through one of the PSQI dimensions [32,38,42]. We found no differences between the control group and the intervention group. However, when analysing only the objective instruments, there was a significant difference in favour of the intervention. Some authors recommend using subjective data as an adjunct to actigraphic data in estimating total sleep time and sleep efficiency in sleep-disordered patients, especially those with disorders of excessive somnolence [62]. Therefore, articles that only report subjective measures should be analysed with caution.

### 4.3. Exercise Programs

Meta-analyses showed very high heterogeneity. This is probably because the populations are very different and range from healthy subjects to older adults with pathologies. However, one aspect that was even more heterogeneous was the intervention. Although most of the programs had a similar frequency (three to five times per week), the duration was highly variable, with programs that lasted between 8 and 12 weeks [36,43,44], up to programs that far exceeded 12 months [35,38,40]. On the other hand, work intensities were not reported in most of the studies. Although it is possible to work at a relatively known intensity in aerobic or strength exercise, it is not so easy to determine the intensity with activities such as pilates, healthy beat acupunch, or yoga, which was used in almost half of the studies [32,36,38,39,42,43].

The most common interventions included were yoga [32,36,38,39,43] and multicomponent exercise [34,40,44]; however, one of the interventions that caught our attention is acupunch [42]. Acupunch is a non-invasive practice where a natural parabola is produced by swinging of the relaxed wrist, elbow, and shoulder joints to direct cuffing and tapping of the fist/palm onto the targeted acupoint along the meridians to transport “qi” [63]. The acupunch itself cannot be considered an exercise or an incentive for physical activity. However, the authors describe the healthy beat acupunch as a modification of the original acupunch by adding the physical fitness guidelines for older adults and essential elements of a comprehensive exercise program for older adults [64].

When analysing by setting, we found that home-based programs (defined as at least 50% of their completion at home) were not effective in improving sleep quality [35,38,40], whereas programs carried out in a facility, such as a nursing home or community centre, were effective if they managed to improve the quality of sleep significantly [32,34,41,43,44]. One of the critical points that could explain the improvement in this type of setting is the degree of supervision. One point in which the programs in facilities agree is the close supervision and guidance in the exercise program [34,44]. Another interesting finding is that when analysing the duration of the programs, those that lasted less than 12 weeks were more effective than those that lasted more than 12 weeks. On the other hand, by adding the analysis by intermediate evaluation points, we were able to find that programs lasting between 12 weeks and 12 months were also effective. However, we believe that it is risky to carry out an analysis since programs older than 12 weeks are few and we think that with the increase in the sample size more conclusive results can be obtained.

An important point to consider is the type of physical training. Although most of the studies comply with the principles of a structured exercise program, with frequency, intensity, time, and duration, as usually happens, physical exercise programs are confused with those that encourage physical activity that consists of increasing moderate to vigorous physical activity (MVPA) [65]. These programs usually increase physical activity through walking. Although both programs show benefits in chronic diseases [65], the principles of physical training cannot be fully applied, so they cannot be analysed as a dose response.

### 4.4. Clinical Implications

This study shows that exercise has effects on sleep quality. Clinicians and decision makers can review these data and carry out exercise programs focused on older adults who report sleep problems with or without pathologies and who report the use of medications for the management of sleep disorders or to improve mental health. This may be particularly relevant at community care levels, such as nursing homes or primary care. On the other hand, it is recommended to carry out supervised programs that comply with the principles of training to achieve the best possible effect on the population.

### 4.5. Limitations

Our study has some limitations. Most of the studies included populations with different diseases or comorbidities, which may limit the extrapolation of the results and recommendations to the entire spectrum of older people, even though our results were statistically significant. The heterogeneous nature of populations implies that many subjects have different pathophysiological behaviours and conditions, a broad spectrum of severity, and the implication or impact that suffering from associated comorbidities may influence the magnitude of reported results. On the other hand, we tried to sub-analyse by health status; however, it was very complex since the definition of healthy is not uniform among the different authors and, therefore, it loses reliability. Additionally, the studies did not report adherence, which is a fundamental factor in determining the effectiveness of an intervention. Future studies must describe the adherence to exercise, since this factor must be analysed together with the results when evaluating the effectiveness of an intervention.

## 5. Conclusions

Physical exercise programs in older adults improve sleep quality, particularly in facilities, and sleep efficiency measured with objective instruments.

## Figures and Tables

**Table 1:** Characteristics of included studies.

Author, Year	Country	Population	Group, *n*	Gender (M/F)	Age (Years)	BMI (kg/m^2^)
Chen et al., 2010	Taiwan	Older adult residents of assisted living facilities	IG: 31	NR	75.4 ± 6.7	NR
			CG: 24	NR		NR
Hirano et al., 2011	Japan	Community-dwelling caregivers	IG: 17	6/11	72.6 ± 4.0	NR
			CG: 14	4/10	75.0 ± 4.6	NR
Oudegeest-Sander et al., 2012	UK	Community healthy older adults	IG: 11	5/6	68 ± 2	25.5 ± 2.1
			CG: 10	5/5	70 ± 2	24.5 ± 2.8
Cheung et al., 2014	USA	Community-dwelling older women with knee osteoarthritis	IG: 11	5/6	71.9 (69.3–74.6) *	29.1 (26.7–31.7) *
			CG: 10	5/5	71.9 (69.0–75.0) *	28.8 (26.0–31.7) *
Ozyemisci Taskiran et al., 2014	Turkey	Older adults living in a nursing home	IG1 (Pilates): 18	3/15	76.2 ± 7.5	29.4 ± 3.5
			IG2 (Yoga): 18	3/15	77.2 ± 6.4	31.5 ± 3.6
			CG: 18	9/13	80.0 ± 6.2	31.0 ± 1.9
Vaz Fragoso et al., 2015	USA	Community-dwelling older adults with mobility limitations	IG: 818	271/547	78.7 ± 5.2	30.1 ± 5.7
			CG: 817	266/551	79.1 ± 5.2	30.3 ± 6.2
Chen et al., 2016	Taiwan	Nursing home older adults in wheelchairs	IG:64	32/32	78.8 ±7.7	NR
			CG: 63	32/31	80.1 ± 6.4	NR
Hsiao et al., 2018	Taiwan	Community-dwelling older adults	IG: 113	20/93	74.7 ± 6.0	NR
			CG: 119	30/89	73.9 ± 5.4	NR
Choi and Sohng, 2018	Republic of Korea	Older adults in community centres	IG: 33	3/30	77.6 ± 5.7	NR
			CG: 30	1/29	78.8 ± 5.8	NR
Laredo-Aguilera et al., 2018	Spain	Community healthy older adults	IG: 20	5/15	75.4 ± 5.3	23.4 ± 17.7
			CG: 18	5/13	76.4 ± 6.5	30.3 ± 3.9
Bademli et al., 2019	Turkey	Older adults living in a nursing home with a mild cognitive impairment	IG: 30	12/18	72.2 ± 7.2	NR
			CG: 30	13/17	70.7 ± 8.3	NR
Falck et al., 2020	Canada	Community older adults with probable mild cognitive impairment	IG: 48	25/23	72 ± 6	26.9 ± 4.2
			CG: 48	16/32	74 ± 5	26.3 ± 4.7
Baklouti et al., 2023	Tunisia	Community-dwelling older adults	IG: 65	42/23	65–75: 71% 76–85: 29%	NR
			CG: 95	56/39	65–75: 78% 76–85: 22%	NR

Abbreviations: BMI: Body mass index; CG: Control group; IG: Intervention group; NR: Not reported. *: Mean (IC95%).

**Table 2:** Characteristics of physical exercise programs.

Author, Year	Type of Exercise	Setting	Duration	Intensity	Frequency	Control Group
Chen et al., 2010	Yoga	Assisted living facilities	6 months	NR	3 times per week	No intervention
Hirano et al., 2011	Walking	Home	12 weeks	Moderate	NR	No intervention
Oudegeest-Sander et al., 2012	Cycling	NR	12 months	70–85% HRR	3 times per week	No intervention
Cheung et al., 2014	Yoga	Home and yoga studio	8 weeks	According to the patient’s performance	5 times per week	Wait list
Ozyemisci Taskiran et al., 2014	G1: Pilates G2: Yoga	Nursing home	8 weeks	According to the patient’s performance	3 times per week	No intervention
Vaz Fragoso et al., 2015	Multicomponent	Home- and centre-based	24–30 months	Moderate	5 times per week	Health education
Chen et al., 2016	Resistance exercise	Nursing home	12 months	Basic (3 m) and advanced (9 m) level	3 times per week	No intervention
Hsiao et al., 2018	Healthy beat acupunch	Community care centre	12 months	NR	3 times per week	Usual activities
Choi and Sohng, 2018	Yoga	Senior community centre	12 weeks	8–14 RPE	4 times per week	Usual care
Laredo-Aguilera et al., 2018	Multicomponent	NR	10 weeks	Self-determined to perform 8–12 rep	3 times per week	Usual activities
Bademli et al., 2019	Multicomponent	Nursing home	20 weeks	Moderate	3–4 times per week	No intervention
Falck et al., 2020	Walking	Home	24 months	Individually tailored	150 min per week	Health enrichment lectures
Baklouti et al., 2023	Yoga	Home	8 weeks	NR	2 times per week	No intervention

Abbreviations: HRR: Heart rate reserve; NR: Not reported; RPE: Rate of perceived exertion.

## Data Availability

Not applicable.
